# Epstein–Barr virus functional RNAs as part of its immune evasion strategy: a role for EBER1?

**DOI:** 10.1098/rsob.250088

**Published:** 2025-11-12

**Authors:** Paul J. Farrell

**Affiliations:** ^1^ Section of Virology, Imperial College London, London, UK

**Keywords:** Epstein–Barr virus, EBER1, EBER2, miRNA, RPL22, exosome

## Abstract

Several types of cancer and some auto-immune diseases are linked to Epstein–Barr virus (EBV) infection. In addition to viral proteins, EBV expresses many functional RNAs to influence cell biology, avoiding the immune responses that would be targeted to viral proteins. Some of these RNAs are also exported from the EBV-infected cells into surrounding cells, including those involved in immune surveillance of the EBV infected cells. EBER1 is the most abundant of these EBV RNAs and recent progress in understanding EBER1 mechanisms might make it a drug target for some diseases associated with EBV.

**Figure:**
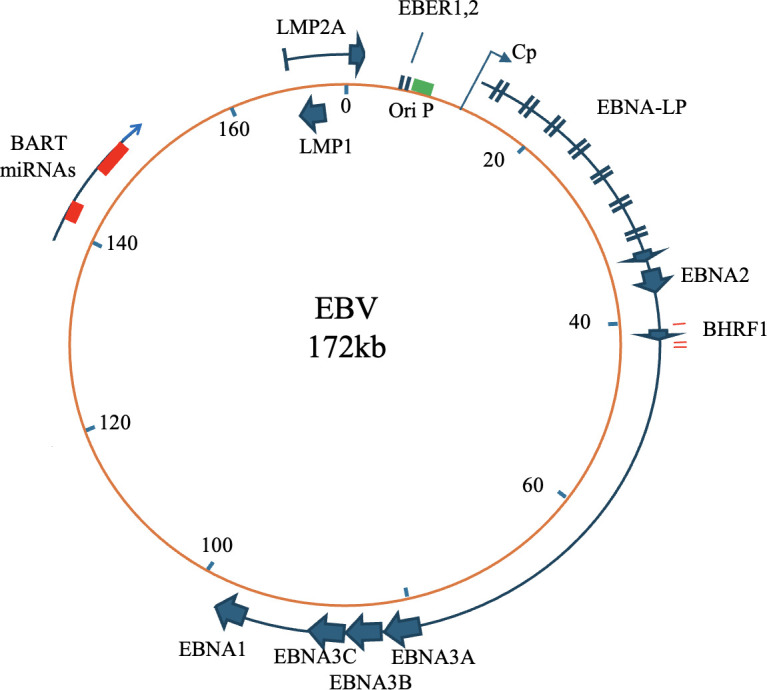

## 1. Epstein–Barr virus is a herpesvirus

Herpesviruses characteristically have two parts to their life cycle [1]. Expression of the virus lytic cycle genes causes replication of the viral double-stranded DNA genome and production of new virus particles. In contrast, the latent cycle genes permit long-term persistence of the virus DNA in certain host cells with no production of infectious virus. The latent virus genomes can persist for the remaining life of the host but may occasionally reactivate into lytic production of infectious virus. Sequences and functions of many of the lytic cycle genes are conserved between different human herpesviruses, in line with the broadly similar lytic viral DNA replication and virus particle assembly. However, the latent cycle genes are quite different, consistent with the different cell types and locations within the body that the various latent herpesviruses occupy. In this sense, the whole family of herpesviruses can be considered as different evolutionary solutions to how to achieve long-term latent persistence in an immune competent host.

The immune evasion mechanisms required for long-term persistence may also differ according to the biological niche in which each virus has evolved to persist. For example, the latent persistence of alpha herpesviruses HSV1, HSV2 and VZV is in specific parts of the nervous system. These are long-lived cells and to some extent immune-privileged sites, good places to hide from immune surveillance. In contrast, major parts of the EBV (a gamma herpesvirus) life cycle and persistence are within the immune system. The virus readily infects B lymphocytes and EBV is thought to persist long-term in a small fraction of our memory B cells [2]. Because it persists in the immune system, its long-term immune evasion requires a strategy suitable for this challenging environment.

## 2. Normal infection by Epstein–Barr virus in healthy people

**Figure 1.**
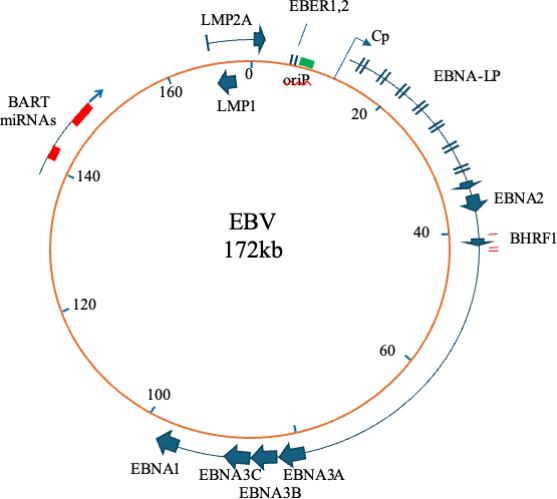
EBV genome map showing genes mentioned in this article. Genes in the lytic replication cycle occupy most of the rest of the genome map, shown in detail in [3].

Most people in the world become infected by EBV in early childhood. It is shed in saliva and can be transmitted by kissing. Transmission is thought to involve transient infection of tonsil epithelium, where the virus can then access B lymphocytes in the tonsil. When EBV infects B lymphocytes, it expresses viral EBNA and LMP proteins (figure 1) that cause the resting B cell to become activated, to proliferate and to migrate to a lymph node, as if the cell had seen its cognate antigen and was participating in a normal immune response. However, it is unlikely that the antigen to which the B-cell receptor on the infected B cell is targeted is being presented in the lymph node; the infected B cell should die but its survival is secured by the EBNAs (which manipulate B cell proliferation) and particularly the LMPs, which prevent apoptosis of the infected cell [4,5]. This allows some of the infected B cells to navigate through the immune system and eventually get into a small fraction of the memory cell population [2]. Here, the host cell is long-lived and only a very low level of EBV EBNA1 protein is required to maintain the viral genome. EBNA1 protein is exceptionally stable, partly mediated by a long gly–ala amino acid repeat sequence which separates the two functional domains of the protein [6]. Because so little viral protein synthesis is needed to maintain the virus in these infected memory cells, there is minimal production of viral peptides that could be presented for immune detection, allowing immune evasion. The virus life cycle can later be completed if the memory cell is activated, eventually migrating to the oral lymphoid organs, where EBV can replicate further in the epithelium and be shed into saliva for transmission.

The result of all this is that the EBV genome persists in some memory B cells, even though there will usually be cytotoxic T lymphocytes (CTLs) that can target many EBV proteins (including EBNA1) in the blood of the infected person. People infected with EBV characteristically have antibodies to EBNA1, but these and the EBNA1 CTLs may be maintained to some extent from EBNA1 protein shed into the blood from EBV infected cells that have died. Initially, the most easily detected CTLs targeting EBNA1 were restricted through MHC class II [7], perhaps suggesting the EBNA1 protein inducing those CTLs was an external antigen.

Many of the EBV proteins involved in virus replication and maintenance also have immune evasion and epigenetic effects that favour persistence of the infection [8–11]. Effects of the latent cycle genes include LMP1 inhibiting interferon responses and LMP2 reducing MHC class II via the CIITA transcription factor [12]. In addition to the tightly controlled expression of viral proteins in latent persistence and the important effects of viral proteins on the host immune responses, EBV also uses many microRNAs (miRNAs) to influence the host cell, avoiding immune responses that would be caused by viral proteins [13,14].

## 3. Functional RNAs expressed by Epstein–Barr virus: miRNAs, EBERs, snoRNA and long non-coding RNAs

The EBER1 and EBER2 genes are adjacent to each other in the EBV genome (figure 1); they are RNA pol III transcripts expressed constitutively during latent infections. EBER1 is 167 nucleotides and EBER2 is 173 nucleotides long. In addition to the EBER RNAs, EBV was one of the first human viruses found to produce miRNAs from viral RNA transcripts [15], which can modulate expression levels of cell mRNAs. The virus can also make a small nucleolar RNA (v-snoRNA1), that may be processed to give miRNA that could regulate the EBV lytic cycle DNA polymerase [16] and two small non-coding RNAs that are expressed in the virus lytic replication cycle have also been described [17].

Long, functional RNAs produced from the EBV genome [18,19] include the BART RNAs (the stable RNA products after splicing out the introns that contain the BART miRNAs) and the BHLF1 and LF3 RNAs, which play roles in the activating the EBV lytic replication origin [20] and are present in the assembling virus particles [21]. BHLF1 and LF3 RNAs from some EBV strains contain a long open reading frame but this is now not thought to be translated because of the highly repetitive amino acid sequence that would be encoded [22]. So, although their sequences do contain some open reading frames, these both appear to function as long non-coding RNAs.

## 4. Epstein–Barr virus miRNAs

Currently 49 EBV miRNAs have been identified [23]; most of them are listed in mIRBase (https://www.mirbase.org/). The miRNAs are excised from non-coding sections of spliced RNA pol II viral transcripts from the BHRF1 region and BART region of the EBV genome (figure 1). Many cell RNAs that can be targeted by EBV miRNAs have been identified and have been reviewed in detail [24–27]. The overall effect is that EBV miRNAs can contribute to inhibition of apoptosis, promotion of cell growth, inhibition of CD4 and CD8 T-cell recognition and inhibition of NK cells. Little is known about EBV miRNA expression in memory B cells, but the miRNAs can generally facilitate EBV persistent infection. They may also contribute to the immune escape that allows growth of cancers containing EBV [28]. There is clear evidence for exosome release of EBV miRNAs to cell culture medium [29] and surrounding cells [24,29,30] which likely plays a role in immune evasion that facilitates the virus persistence.

Although EBV persists in memory B cells and only infects B cells in adult blood, some T cells in new-born children and young infants can also be infected by EBV [31,32]. Unlike adult T cells, these T cells express the EBV receptor CR2, also called CD21 [33]. The extent to which T cells normally become infected by EBV in healthy children may be low but there are various childhood T-cell lymphomas that contain EBV. Interestingly, the EBV in these lymphoma cells often has a deletion of part of the BART miRNA locus, even though the main EBV infection of B cells in the same people contains a wild-type EBV genome. This suggests that loss of some BART miRNA functions may permit the cancer development [23,32].

## 5. EBER2 can promote cell proliferation

EBER2 is constitutively expressed in all EBV latent infections and is the second most abundant RNA transcript from the viral genome. Early investigations of EBER2 function by mutation of lab strains of EBV seemed to give conflicting results. Mutating EBER2 in the B95-8 strain of EBV was found to have no significant effect on immortalization or subsequent growth of the transformed lymphoblastoid cell lines (LCLs) [34] but similar experiments in Akata EBV indicated a role for EBER2 in LCL growth [35]. That strain difference in phenotype is now thought to have been due partly to the very strong signalling from the B95-8 LMP1 masking the EBER2 effect in the B95-8 experiments. An important clue to a role of EBER2 *in vivo* came from studies comparing EBV in Chinese nasopharyngeal carcinoma (NPC) cases with healthy Cantonese people. This linked a sequence polymorphism in EBER2 to NPC [36,37]. The Akata EBV genetics and the NPC data implied a function in cell growth or survival, but it has taken several years for this to be understood.

EBER2 is now known to form a ribonucleoprotein complex in the cell nucleus that affects specific gene expression via PU.1 and other transcription factors. This was first described to affect the transcription of the EBV LMP2 gene via binding to the EBV terminal repeats [38] but further analysis has shown that it also increases expression of the cell UCHL1 gene [39]. Base pairing between part of the EBER2 RNA and the 5′-end of the nascent UCHL1 mRNA releases transcription pausing and facilitates expression of the UCHL1 mRNA. UCHL1 is a de-ubiquitinase that increases the expression of cyclin B1 and Aurora B; both are cell cycle control proteins. EBER RNAs with the Chinese M81 variant of EBER2 (which has a sequence change that increases its binding efficiency to the nascent UCHL1 mRNA) resulted in about fivefold more Aurora B protein expression than the reference EBER genes [39]. There is therefore a role for EBER2 in promoting cell cycle progression and cell growth of cells infected by EBV, potentially stronger in the EBV found in NPC cases.

## 6. EBER1 is expressed in Epstein–Barr virus infected memory cells and Epstein–Barr virus cancer cells

EBER1 is the most abundant EBV RNA. It is located mainly in the cell nucleoplasm and its expression has been demonstrated directly in memory B cells of healthy EBV carriers by RT-PCR [40] and by flow cytometry (C. Shannon-Lowe, personal communication, 2025). However, deletion of EBER1 from EBV in the reference B95-8 strain background seemed to make no significant difference to the ability of the virus to transform human B cells into LCLs, growth of the LCLs [34,41], sensitivity to interferon treatment [41] or establishing persistent infection in humanized mice [42]. At first, it was puzzling that the most abundant gene product of the virus did not seem to have an obvious function in EBV infected cells. One clue for an *in vivo* function came from finding that EBER1 could substitute for non-coding RNA TMER4 of murine herpesvirus 68 (MHV68, a gamma herpesvirus of mice) in MHV68 infected mice. It could maintain egress of MHV68-infected B cells from lymph nodes into the peripheral circulation, which was otherwise reduced in the absence of TMER4 [43]. This suggested a role for EBER1 in fully immune competent people, which was perhaps not apparent in cell culture or the humanized mice that had been studied previously.

## 7. EBER1 binds the cell La protein and ribosomal protein RPL22

After the identification of EBER1 in 1981 [44], it was found to bind efficiently to the cell La protein [45,46] and to ribosomal protein RPL22 [47]. In other RNA pol III transcripts such as tRNAs and 5S RNA, the 3′-oligo U-termination sequence is eventually processed away but it remains on EBER1 within the EBV-infected cell [45,46] and EBER1 also retains its 5′ triphosphate, which can be recognized by RIG-I [48]. The folded EBER RNAs contain some dsRNA segments, which can activate signalling from TLR3 in infected cells [49] and even the level of EBER RNAs packaged within the virion is sufficient to induce interferon production upon infection of B cells by EBV [50].

Although La is well known to assist in folding and maturation of RNA Pol III nascent transcripts in the nucleus [51], cytoplasmic La affects the expression of certain classes of mRNA that have a 5′-oligopyrimidine (5′-TOP) sequence [52] and can also bind to the polyA tail of mRNA [53] to control protein synthesis. Additional novel functions of La protein in regulating the development of osteoclasts [54] demonstrate its complex biological roles and suggest potential off target effects that might result from a drug that attempted to block EBER1 interaction with La.

For many years, the significance of the EBER1 binding to RPL22 remained a puzzle; how could an important ribosomal protein be mostly sequestered in this way, when EBV infected cells proliferate so well? This RPL22 question was clarified by an impressive series of mouse experiments using knockout of the RPL22 gene to study its function. It is now understood [55] that there is an additional cell gene involved, called L22L1. This gives a protein product (L22L1) that is a paralogue of RPL22 and can substitute for RPL22 in protein synthesis. So, deletion of the RPL22 gene does not prevent protein synthesis or growth of most cell types and RPL22−/− mice were found to develop similarly to wild-type mice. Normally, RPL22 protein inhibits L22L1 expression by binding to an internal hairpin structure in the L22L1 mRNA [55], which is why L22L1 had not been detected previously.

## 8. EBER1 may mediate immune evasion and provide a therapeutic target

The improved understanding of RPL22 and L22L1 was an important step but at first it was not clear how this would relate to EBV biology and EBER1. In mice, RPL22 and L22L1 can both influence haematopoiesis via Smad1; L22L1 binds and increases translation of Smad1 mRNA, but RPL22 represses Smad1 translation [56]. Additionally, induction of L22L1 caused by expression of EBER1 results in an increased level of oxidative phosphorylation in the cells [57] and RPL22 protein has also been found to bind specifically to human telomerase RNA [58].

No effect of RPL22 deletion was observed on breeding of mice and generation of offspring in a clean animal house in the absence of infection [55] but further analysis of the RPL22−/− mice revealed much lower levels of αβ T lymphocytes, including cytotoxic T cells [59]. This results from activation of a p53-dependent checkpoint that selectively blocks development of precursors of αβ T cells. The levels of γδ T cells were not affected by RPL22 deletion in wild-type mice [59]; and in p53 null mice, there was no effect of RPL22 deletion on T-cell levels. The mechanism seems to involve RPL22 normally restraining endoplasmic reticulum (ER) stress responses in αβ T cells, which in the absence of functional RPL22 can induce p53 and cell growth arrest [60]. In cell culture, antigen-specific proliferative responses of CD4+ αβ T cells are known to require downregulation of p53. Avoiding the p53 response allows αβ T-cell proliferation and the mechanism helps to enforce antigen specificity of the proliferating T cells [61], avoiding autoimmunity.

The mouse observations on loss of RPL22 function causing growth arrest of CD4+ αβ T cells were an important step forward but did not immediately explain what the value of expressing high levels of EBER1 in B lymphocytes would be (since the mouse phenotype was in T lymphocytes). Investigation of the function of EBER1 had mostly focussed on the properties of EBV-infected cells but some EBER1 is also exported from EBV infected cells in exosomes [48,50,62]. EBER1 released from EBV-infected cells has been shown to activate signalling by TLR3 in cells that take up the EBER1 [49] and a role for secreted EBER1 has been proposed in some auto-immune diseases [48,49]. The explanation for the paradox that EBER1 is so abundant but has little effect in the EBV infected B cell may therefore be that the real function of EBER1 is to migrate into surrounding T cells and prevent their function—a form of immune evasion that could aid persistence of the virus *in vivo* and in EBV diseases. This role of EBER1 would be analogous to the known export of miRNAs in exosomes [30]. Since normal EBV carriers are not generally immunosuppressed, this would be a local effect in T cells stably around the EBV-infected cells, either in normal carriers or in an EBV-associated cancers.

## 9. Secreted EBER1 as a target for therapy?

**Figure 2.**
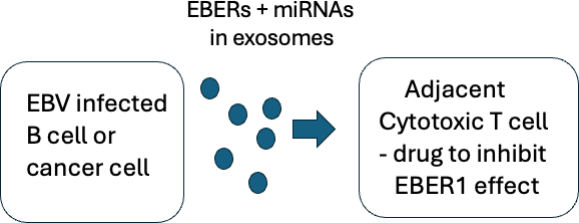
EBV RNA functional RNAs affect T-cell function and could be a drug target. EBV infected cell secretes exosomes containing EBERs and EBV miRNAs, suppressing adjacent T-cell function. It would be difficult to specifically target the large number of different EBV miRNAs but a drug that inhibited EBER1 binding to RPL22 might reduce this mechanism of immune evasion. L22 deficiency in mice is known to result in p53-dependent absence of αβ T cells [59].

An interesting potential example of this exported EBER1 effect might be in NPC. Small patches of the malignant EBV-infected epithelial cells (which express high levels of EBER1) are characteristically interspersed with areas of lymphoid cells, which do not seem to mount an effective immune response to the EBV infected cells. Boosting immune responses by immunization with EBV proteins generally had less effect on NPC cancers than might be expected from the impressive increase in antibodies and circulating EBV-specific T cells that was achieved [37,63]. Perhaps local export of EBER1 from the EBV-infected cancer cells is sufficient to bind RPL22 in adjacent cytotoxic αβ T cells that are present in the lymphocytic infiltrate and consequently prevent survival or function of these T cells, contributing to immune evasion by the cancer. Transfer of EBER1 to cytotoxic T cells could thus mediate immune evasion in natural persistence and in cancers containing EBV. Since standard lab cytotoxic T-cell assays work well on EBV LCLs that contain EBER1, this might be a feature of the stable, long-term interaction of cells in a cancer. If this mechanism proves to be quantitatively significant, drugs that could block export of the EBER1 or this function of EBER1 in the T cells might provide a novel approach to therapy for cancers associated with EBV (figure 2). Specificity might be achieved by screening for compounds that could inhibit the interaction of EBER1 with RPL22 but not the other binding partners of EBER1 or normal function of RPL22. This EBER1 targeted drug approach might be more feasible than attempting to inhibit the many different exported EBV miRNAs, potentially making EBER1 an attractive drug target.

## Data Availability

This article has no additional data.
